# Pediatric Sleep-Disordered Breathing and Long-Term Complications: Clinical and Health Implications

**DOI:** 10.3390/jcm11175178

**Published:** 2022-09-01

**Authors:** Marco Zaffanello, Massimo Franchini, Giorgio Piacentini

**Affiliations:** 1 Department of Surgical Sciences, Dentistry, Gynecology and Pediatrics, University of Verona, 37126 Verona, Italy; 2 Department of Hematology and Transfusion Medicine, Carlo Poma Hospital, 46100 Mantova, Italy

Transitional medicine is defined as the branch of medicine which deals with the transition from the pediatric to adult healthcare system. The transitions in healthcare must be individualized and coordinated through a collaborative process between pediatric and adult health professionals [1,2]. Transitional medicine for chronic diseases is a challenge to the process of continuous and structured healthcare for all ages of life.

Obstructive sleep apnea syndrome (OSAS) is a sleep breathing disorder characterized by repeated episodes of partial or complete obstruction of the upper respiratory tract. This obstruction usually occurs with a partial or complete reduction in airflow in the upper airways accompanied by a respiratory effort. The prevalence of sleep disordered breathing (SDB) has been reported in the range of 2 to 11% [3] in pediatric populations and between 2 and 4% of adult populations [4].

Various patterns of SDB have been described in children, suggesting that distinct clinical phenotypes may exist. The size of the tonsils and adenoids was associated with the severity of OSAS in pre-school and school children and laryngomalacia and gastroesophageal reflux in the younger group [5]. The obese phenotype associated with SDB is more frequent in adolescence [6]. Finally, some genetic diseases (Down syndrome, Prader–Willi syndrome, etc.) show increased risk of SDB. In these children, the early diagnosis and treatment of SDB reduces organ damage [7] and improves quality of life [8,9]. In adults, the main cause of SDBs is obesity. Sleep bruxism, snoring, and excessive daytime sleepiness are common in adults and appear to be associated with a higher incidence of severe OSAS [10].

In mild forms of pediatric OSAS, secondary to adenotonsillar hypertrophy and allergies, treatment is in first instance conservative by medical therapy (nasal steroids, leukotriene receptor antagonists). In severe pediatric OSAS, the first therapeutic choice is adenotonsillectomy. Another important treatment option is the rapid expansion of the upper jaw. Myofunctional therapy is useful to associate orthodontic therapy, although in adults it has shown low adherence [11]. The mandibular advancement device is able to significantly increase the three-dimensional size of the upper airways [12]. Allergy therapy, cPAP [13] and, in obese children and adults, weight control are other treatment options. A detailed understanding of the complex pathophysiology of OSA encourages the development of therapies targeting pathophysiological endotypes and facilitates the transition to precision medicine [14].

OSAS is frequently associated with metabolic, cardiovascular, renal, pulmonary, and neuropsychiatric comorbidities. OSAS causes growth retardation in children by showing significant recovery after adenotonsillectomy, as demonstrated by changes in the levels of IGF-1, IGFBP-3, ghrelin, and leptin [15]. OSAS leads to adverse cardiovascular sequelae, and the long-term consequences may include endothelial dysfunction, oxidative stress, and the up-regulation of inflammatory cytokines, ultimately leading to a variety of neurological and cardio-vascular complications [15,16,17]. In pediatric ages, OSAS triggers a process of vascular endothelial damage similar to a pre-atherosclerotic condition [17]. Dyslipidemia is well known in OSAS and could contribute to the development of cardiovascular diseases [18]. OSAS is highly prevalent among patients with asymptomatic left ventricular systolic and diastolic dysfunction and congestive heart failure [19]. The incidence of atrial fibrillation is 88% higher in patients with OSAS [4].

Pediatric obesity, like adults, has reached epidemic levels in Westernized societies. Obesity is difficult to treat. A systematic review showed that obese children and adolescents were about five times more likely to be obese in adulthood than those who were not obese. In addition, more than half of obese adults were not obese during childhood or adolescence [20]. Organ damage caused by OSAS begins as early as pediatric age and may persist and worsen in adulthood. In particular, in children, the association of obesity and OSAS increases the risk of cardiovascular morbidity in adulthood [18,21,22]. Children with severe OSAS have an increased risk of suffering from snoring, overweight, lower academic achievement, and chronic diseases once they grow into adults [23].

It is well known that adults with OSAS have an increased risk of eye disease. Recently, it has been shown that children with OSAS have eye disorders. This would suggest that ocular comorbidities could begin in the child with OSAS and persist and worsen in later ages [24].

During the ongoing COVID-19 pandemic, the delay in the diagnosis and treatment of OSAS is causing important health consequences at all ages [25]. In fact, the intervening pandemic of the COVID-19 disease has introduced additional neurobehavioral morbidity that complicates the identification of the long-term consequences of childhood OSAS [23].

It is necessary to strengthen educational programs aimed at increasing the awareness, diagnosis, and management of SDB starting from the pediatric age in order to ensure efficient management and reduce the health burden of the disease [26] for both the patient and the healthcare system.

As the incidence of pediatric patients at high risk of OSAS increases, a growing number of young adults will require long-term medical transition follow-up. Heffernan et al. reported that a transition program has been implemented in Canada for some years in adolescent patients with OSAS [27]. The adequate training of healthcare professionals and the implementation of transition programs are essential. The intrinsic chronicity of pathologies at the risk of persistent SDB requires the activation of transitional care pathways from pediatric to adult medicine. Pediatricians and pediatric associations should work together with adult medicine to carry out such transition in an increasingly structured way.

## References

[1] Roberts G., Vazquez-Ortiz M., Knibb R., Khaleva E., Alviani C., Angier E., Blumchen K., Comberiati P., Duca B., DunnGalvin A. (2020). EAACI Guidelines on the Effective Transition of Adolescents and Young Adults with Allergy and Asthma. Allergy.

[2] Kumagai H., Shimizu T., Iwama I., Hagiwara S.-I., Kudo T., Takahashi M., Saito T., Kunisaki R., Uchino M., Hiraoka S. (2022). A Consensus Statement on Health-Care Transition for Childhood-Onset Inflammatory Bowel Disease Patients. Pediatr. Int. Off. J. Jpn. Pediatr. Soc..

[3] Di Carlo G., Zara F., Rocchetti M., Venturini A., Ortiz-Ruiz A.J., Luzzi V., Cattaneo P.M., Polimeni A., Vozza I. (2020). Prevalence of Sleep-Disordered Breathing in Children Referring for First Dental Examination. A Multicenter Cross-Sectional Study Using Pediatric Sleep Questionnaire. Int. J. Environ. Res. Public Health.

[4] Moula A.I., Parrini I., Tetta C., Lucà F., Parise G., Rao C.M., Mauro E., Parise O., Matteucci F., Gulizia M.M. (2022). Obstructive Sleep Apnea and Atrial Fibrillation. J. Clin. Med..

[5] Nosetti L., Zaffanello M., De Bernardi F., Piacentini G., Roberto G., Salvatore S., Simoncini D., Pietrobelli A., Agosti M. (2020). Age and Upper Airway Obstruction: A Challenge to the Clinical Approach in Pediatric Patients. Int. J. Environ. Res. Public Health.

[6] Jennum P., Ibsen R., Kjellberg J. (2013). Morbidity and Mortality in Children with Obstructive Sleep Apnoea: A Controlled National Study. Thorax.

[7] Betavani V.M.P., Davey M.J., Nixon G.M., Walter L.M., Horne R.S.C. (2022). Effects of Treatment of Sleep Disordered Breathing on Sleep Macro- and Micro-Architecture in Children with Down Syndrome. Children.

[8] Oros M., Baranga L., Plaiasu V., Cozma S.R., Neagos A., Paduraru L., Necula V., Martu C., Dima-Cozma L.C., Gheorghe D.C. (2021). Obstructing Sleep Apnea in Children with Genetic Disorders-A Special Need for Early Multidisciplinary Diagnosis and Treatment. J. Clin. Med..

[9] Wong S.-B., Yang M.-C., Tzeng I.-S., Tsai W.-H., Lan C.-C., Tsai L.-P. (2022). Progression of Obstructive Sleep Apnea Syndrome in Pediatric Patients with Prader-Willi Syndrome. Children.

[10] Smardz J., Wieckiewicz M., Wojakowska A., Michalek-Zrabkowska M., Poreba R., Gac P., Mazur G., Martynowicz H. (2022). Incidence of Sleep Bruxism in Different Phenotypes of Obstructive Sleep Apnea. J. Clin. Med..

[11] O’Connor-Reina C., Ignacio Garcia J.M., Rodriguez Alcala L., Rodríguez Ruiz E., Garcia Iriarte M.T., Casado Morente J.C., Baptista P., Plaza G. (2021). Improving Adherence to Myofunctional Therapy in the Treatment of Sleep-Disordered Breathing. J. Clin. Med..

[12] Camañes-Gonzalvo S., Marco-Pitarch R., Plaza-Espín A., Puertas-Cuesta J., Agustín-Panadero R., Fons-Font A., Fons-Badal C., García-Selva M. (2021). Correlation between Polysomnographic Parameters and Tridimensional Changes in the Upper Airway of Obstructive Sleep Apnea Patients Treated with Mandibular Advancement Devices. J. Clin. Med..

[13] Tondo P., Pronzato C., Risi I., D’Artavilla Lupo N., Trentin R., Arcovio S., Fanfulla F. (2022). Switch of Nocturnal Non-Invasive Positive Pressure Ventilation (NPPV) in Obstructive Sleep Apnea (OSA). J. Clin. Med..

[14] McNicholas W.T., Pevernagie D. (2022). Obstructive Sleep Apnea: Transition from Pathophysiology to an Integrative Disease Model. J. Sleep Res..

[15] Zaffanello M., Piacentini G., La Grutta S. (2020). Beyond the Growth Delay in Children with Sleep-Related Breathing Disorders: A Systematic Review. Panminerva Med..

[16] Maniaci A., Iannella G., Cocuzza S., Vicini C., Magliulo G., Ferlito S., Cammaroto G., Meccariello G., De Vito A., Nicolai A. (2021). Oxidative Stress and Inflammation Biomarker Expression in Obstructive Sleep Apnea Patients. J. Clin. Med..

[17] Zolotoff C., Bertoletti L., Gozal D., Mismetti V., Flandrin P., Roche F., Perek N. (2021). Obstructive Sleep Apnea, Hypercoagulability, and the Blood-Brain Barrier. J. Clin. Med..

[18] Zaffanello M., Piacentini G., La Grutta S. (2020). The Cardiovascular Risk in Paediatrics: The Paradigm of the Obstructive Sleep Apnoea Syndrome. Blood Transfus..

[19] Javaheri S., Javaheri S. (2022). Obstructive Sleep Apnea in Heart Failure: Current Knowledge and Future Directions. J. Clin. Med..

[20] Simmonds M., Llewellyn A., Owen C.G., Woolacott N. (2016). Predicting Adult Obesity from Childhood Obesity: A Systematic Review and Meta-Analysis. Obes. Rev. Off. J. Int. Assoc. Study Obes..

[21] Pardak P., Filip R., Woliński J. (2022). The Impact of Sleep-Disordered Breathing on Ghrelin, Obestatin, and Leptin Profiles in Patients with Obesity or Overweight. J. Clin. Med..

[22] Torres-Lopez L.V., Cadenas-Sanchez C., Migueles J.H., Adelantado-Renau M., Plaza-Florido A., Solis-Urra P., Molina-Garcia P., Ortega F.B. (2020). Associations of Sedentary Behaviour, Physical Activity, Cardiorespiratory Fitness and Body Composition with Risk of Sleep-Related Breathing Disorders in Children with Overweight/Obesity: A Cross-Sectional Study. J. Clin. Med..

[23] Nosetti L., Zaffanello M., Katz E.S., Vitali M., Agosti M., Ferrante G., Cilluffo G., Piacentini G., Grutta S.L. (2022). Twenty-Year Follow-up of Children with Obstructive Sleep Apnea. J. Clin. Sleep Med..

[24] Bonacci E., Fasolo A., Zaffanello M., Merz T., Brocoli G., Pietrobelli A., Clemente M., De Gregorio A., Longo R., Bosello F. (2022). Early Corneal and Optic Nerve Changes in a Paediatric Population Affected by Obstructive Sleep Apnea Syndrome. Int. Ophthalmol..

[25] Bikov A., Khalil S., Gibbons M., Bentley A., Jones D., Bokhari S. (2021). A Fully Remote Diagnostic and Treatment Pathway in Patients with Obstructive Sleep Apnoea during the COVID-19 Pandemic: A Single Centre Experience. J. Clin. Med..

[26] Nosetti L., Paglietti M.G., Brunetti L., Masini L., La Grutta S., Cilluffo G., Ferrante G., Zaffanello M., Verrillo E., Pavone M. (2020). Application of Latent Class Analysis in Assessing the Awareness, Attitude, Practice and Satisfaction of Paediatricians on Sleep Disorder Management in Children in Italy. PLoS ONE.

[27] Heffernan A., Malik U., Cheng R., Yo S., Narang I., Ryan C.M. (2019). Transition to Adult Care for Obstructive Sleep Apnea. J. Clin. Med..

